# α-Lithiation and Electrophilic Substitution of 1,4,4-Trimethyl-3,4-dihydroquinolin-2-one

**DOI:** 10.3390/molecules15117742

**Published:** 2010-10-29

**Authors:** Bouclé Sébastien, Guillard Jérôme, Viaud-Massuard Marie-Claude

**Affiliations:** UMR CNRS 6239 GICC, Laboratoire de Chimie Organique & Thérapeutique, UFR des Sciences Pharmaceutiques, Université François Rabelais de Tours, 31 avenue Monge, 37200 Tours, France

**Keywords:** tetrahydroquinoline, electrophilic substitution, anti-diabetic agents

## Abstract

Treatment of 1*,*4,4-trimethyl-3,4-dihydroquinolin-2(1*H*)-one (**2**) with lithium diisopropylamide (LDA) followed by a wide range of electrophiles give the corresponding 4,4-dimethyl-3-substituted-3,4-dihydroquinolin-2-ones **3**-**13**, providing a very mild electrophilic substitution of the 4,4-dimethyl-1,2,3,4-tetrahydroquinoline core.

## 1. Introduction

**Figure 1:**
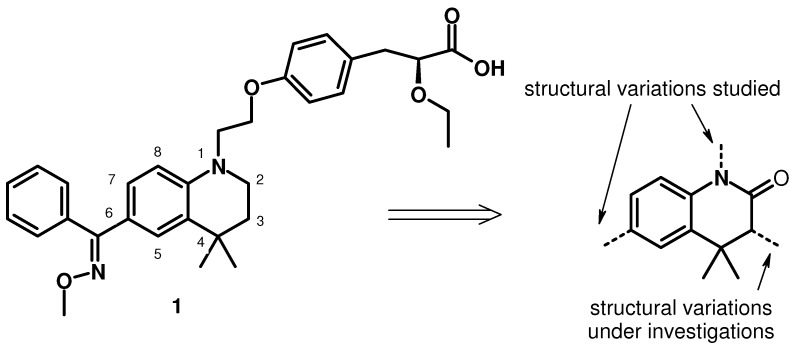
Lead compound **1**.

The chemistry of 4,4-dimethyl-3,4-dihydroquinolin-2-one has been the focus of interest of many investigations during recent years. This growing interest can be explained because quinolinone derivatives are an important class of natural product exhibiting a broad spectrum of biological activities. Moreover, substituted or reduced 4,4-dimethyl-3,4-dihydroquinolin-2-ones are the core structures of many pharmacological agents and drug molecules such as anti-cancer drugs [1,2], anti-diabetic [3,4,5] or cardiovascular agents [6], pesticides [7,8] and active components of various dyes [9,10,11]. Consequently, synthetic methodologies for preparing dihydroquinolin-2-one derivatives have attracted considerable interest, and several methods offering good results have been reported [3,4,5,6,7,8,9,10,11,12]. The nature, number and relative location of the substituents are the key parameters to consider before choosing a particular method, however, few methods have been investigated with the aim of obtaining 4,4-dimethyl-3-substituted-3,4-dihydroquinolin-2-ones. We have recently reported the design, synthesis and biological data of a novel 4,4-dimethyl-1,2,3,4-tetrahydroquinoline-based series of PPARγ partial agonists and PPARα/γ dual agonists [3,4]. This study has led to the identification and optimization of our lead compound **1** (Figure 1), which has noteworthy dual-activity on both subtypes.

Our lead compound **1** possesses an acid head, α-ethoxy-β-phenylpropionic acid moiety, which is a potent binding moiety for both PPARα and PPARγ, and a cyclic tail, consisting of the 4,4-dimethyl-1,2,3,4-tetrahydroquinoline skeleton that tolerates more polar substituents. In order to investigate the impact of changes on the transactivation activity, we decided to substitute position 3 of this cyclic tail.

## 2. Results and Discussion

**Scheme 1:**
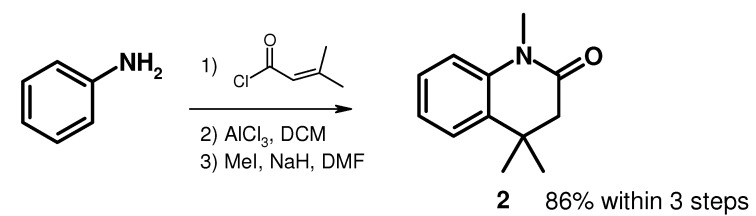
Synthesis of 4,4-trimethyl-3,4-dihydroquinolin-2(1*H*)-one (**2**).

Thus, our strategy was to carry out an α-lithiation and an electrophilic substitution on 1,4,4-trimethyl-3,4-dihydroquinolin-2-one (**2**). This compound was prepared according to the synthetic sequence outlined in Scheme 1 [6]. Aniline was treated with 3,3-dimethylacryloyl chloride to give the corresponding amide, which was cyclized under Friedel-Crafts conditions and then methylated.

**Scheme 2:**
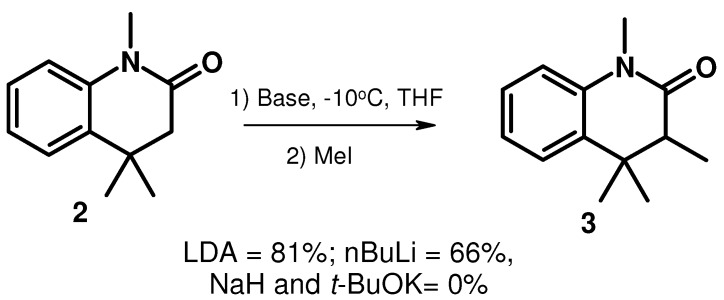
Synthesis of compound **3**.

We then tried to lithiate 1*,*4,4-trimethyl-3,4-dihydroquinolin-2(1*H*)-one with 1 equiv of *n*-BuLi or LDA in THF at −10 °C (Scheme 2). The α-anion thus obtained was condensed with methyl iodide, providing the 3-methyl product in yields of 66% and 81%, respectively. When sodium hydride or potassium *tert*-butoxide was used as the strong base, no reaction was observed. 

Consequently a wide range of electrophiles have been assayed in this reaction, and the resulting products are summarized in Table 1. Most of these electrophilic substitutions gave good yields with no detectable secondary substitution.

**Table 1:** Lithiation and Electrophilic Substitution of **2**. 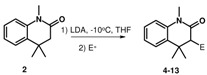

Entry	E^+^	E	Product^a^	Yield^b^ (%)
1	EtI	Et	4	94
2	BnBr	Bn	5	99
3	Allyl-Br	Allyl	6	93
4	Br(CH_2_)_5_-Br	(CH_2_)_5_-Br	7	60
5	PhCHO	CH(OH)Ph	8	99
6	CH_3_-CO-Ph	CH_3_-CH(OH)-Ph		-^c^
7	I_2_	I	9	88
8	Ac_2_O	Ac	10	68
9	PhSO_2_Cl	Cl	11	90^d^
10	PhSO_2_F	PhSO_2_	12	46
11	CO_2_	COOH	13	75
12	B(O- *i*Pr)_3_	B(OH)_2_		-^c^

^a^: all compounds were characterized by IR, NMR and GCMS. ^b^: Yield of isolated material. ^c^: no reaction. ^d^: only chlorinated product was isolated.

Reactions with halogenoalkanes (entries 1-4) provide an access to alkyl derivatives with excellent yields. Alkylation using dibromoalkane provided a moderate yield of the target product and a cross-coupling product. In the case of carbonyl derivatives (entries 5, 6, 8 and 11), only ketone did not react with lithiated species. With sulfonyl compounds as the electrophile, we obtained two different products, depending on the nature of the anion in the sulfonyl group. The target chlorinated product **11** (entry 9) was synthesized using benzenesulfonyl chloride [13,14,15]. However, using benzenesulfonyl fluoride provided the corresponding sulfonyl product **12** with moderate yield, and unreacted starting compound **2**. 

## 3. Experimental

### 3.1. General

All reactions requiring anhydrous conditions were conducted in flame dried apparatus under an atmosphere of argon. THF was freshly distilled from benzophenone-sodium. All reagents and starting materials were purchased from commercial sources and used as received. All lithiations were carried out using 2.0 M LDA in tetrahydrofuran/heptane/ethylbenzene (Sigma-Aldrich). Analytical TLC was carried out on silica gel F_254_ plates. Visualization was achieved by UV light (254 nm). Flash column chromatography was carried using Sigma-Aldrich Versaflash silica gel (particle size 20–45 µm). Melting points (m.p.) were measured on a Büchi B-540 capillary melting point apparatus and are uncorrected. ^1^H- and ^13^C-NMR spectra were recorded in CDCl_3_ using a Bruker Avance 300 (operating frequencies: ^1^H, 300.13 MHz; ^13^C, 75.47 MHz) FT spectrometer at ambient temperature. The chemical shifts (δ, ppm) for all compounds are listed in parts per million downfield from tetramethylsilane using the NMR solvent as an internal reference. The reference values used for deuterated chloroform (CDCl_3_) were 7.26 and 77.00 ppm for ^1^H- and ^13^C-NMR spectra, respectively. Multiplicities are given as: s (singlet), brs (broad singlet), d (doublet), t (triplet), dd (doublet of doublets), td (triplet of doublets) or m (multiplet). Low-resolution mass (MS) were recorded on a Shimadzu GCMS-QP 2010 Gas Chromatograph Mass Spectrometer and reported in units of mass to charge (m/z). The mode of ionization used was electron-impact (EI). 

### 3.2. General procedure

To a stirring solution of **2** (0.1 g, 0.53 mmol) in THF (5 mL) cooled at −10 °C under an argon atmosphere was added 0.275 mL of LDA (1.3 equiv., 0.689 mmol, 2.0 M in tetrahydrofuran/heptane/ ethylbenzene). After completion of the addition, the mixture was stirred for 30 min at −10 °C, and then the appropriate electrophile (1.2 equiv, 0.636 mmol) was added. The reaction mixture was stirred at −10 °C for 2h and allowed to warm slowly to room temperature before being added dropwise to a saturated aqueous solution of ammonium chloride (80 mL) and extracted with ethyl acetate (3 × 40 mL), the combined organic layers were dried over anhydrous magnesium sulfate, and concentrated *in vacuo* to afford the crude products, which were purified by column chromatography with cyclohexane-ethyl acetate as the eluant.

*1,3,4,4-Tetramethyl-3,4-dihydroquinolin-2(1H)-one* (**3**): Yield 86%; white oil; ^1^H-NMR (CDCl_3_) δ: 1.04 (3H, d, *J* = 7.2 Hz, CH_3_), 1.22 (3H, s, CH_3_), 1.25 (3H, s, CH_3_), 2.44 (1H, q, *J* = 7.2 Hz, CH), 3.38 (3H, s, NCH_3_), 7.01 (1H, d, *J* = 8.1 Hz, H_ar_), 7.07 (1H, td, *J* = 7.5, 1.2 Hz, H_ar_), 7.26–7.30 (2H, m, 2 × H_ar_); ^13^C-NMR (CDCl_3_) δ: 11.4 (CH_3_), 22.8 (CH_3_), 27.6 (CH_3_), 29.6 (CH_3_), 35.8 (C), 47.3 (CH), 114.8 (CH), 123.3 (CH), 124.8 (CH), 127.3 (CH), 133.9 (C), 138.8 (C), 173.1 (C); MS (EI) m/z 188, 100% [M-CH_3_^+^], 203, 55% [M^+^]; Calcd for C_13_H_17_NO: C, 76.81; H, 8.43; N, 6.89. Found: C, 76.26; H, 8.41; N, 6.82. 

*3-Ethyl-1,4,4-trimethyl-3,4-dihydroquinolin-2(1H)-one* (**4**): Yield 94%; whitish solid; Mp 72–77 °C; ^1^H-NMR (CDCl_3_) δ: 0.92 (3H, t, *J* = 7.2 Hz, CH_3_), 1.07–1.18 (1H, m, CH), 1.21 (3H, s, CH_3_), 1.35 (3H, s, CH_3_), 1.49-1.63 (1H, m, CH), 2.24 (1H, dd, *J* = 10.8, 4.5 Hz, CH_2_), 3.40 (3H, s, NCH_3_), 7.00 (1H, d, *J* = 8.4 Hz, H_ar_), 7.07 (1H, td, *J* = 7.5, 1.2 Hz, H_ar_), 7.25 (2H, m, 2 × H_ar_); ^13^C-NMR (CDCl_3_) δ: 12.5 (CH_3_), 19.7 (CH_2_), 23.4 (CH_3_), 28.9 (CH_3_), 29.4 (CH_3_), 36.1 (C), 55.3 (CH), 114.7 (CH), 123.2 (CH), 124.8 (CH), 127.2 (CH), 133.9 (C), 138.9 (C), 172.1 (C); MS (EI): m/z 174, 100% [M-CH_3_-CH_2_CH_3_^+^], 217, 62% [M^+^]; Calcd for C_14_H_19_NO: C, 77.38; H, 8.81; N, 6.45. Found: C, 77.90; H, 8.61; N, 6.52. 

*3-Benzyl-1,4,4-trimethyl-3,4-dihydroquinolin-2(1H)-one* (**5**): Yield 99%; yellowish solid; Mp 46–51 °C; ^1^H-NMR (CDCl_3_) δ: 1.32 (3H, s, CH_3_), 1.42 (3H, s, CH_3_), 2.64 (1H, dd, *J* = 12.8, 8.9 Hz, CH), 2.73 (1H, dd, *J* = 8.9, 4.5 Hz, C**H_a_**H_b_Ph), 2.86 (1H, dd, *J* = 12.9, 4.5 Hz, CH_a_**H_b_**Ph), 3.37 (3H, s, NCH_3_), 7.03 (1H, d, *J* = 8.1 Hz, H_ar_), 7.1–7.35 (8H, m, 8 × H_ar_); ^13^C-NMR (CDCl_3_) δ: 23.6 (CH_3_), 28.4 (CH_3_), 29.6 (CH_3_), 33.1 (CH_2_), 36.6 (C), 55.2 (CH), 114.9 (CH), 123.3 (CH), 124.9 (CH), 126.2 (CH), 127.5 (CH), 128.2 (2xCH), 128.9 (2xCH), 133.8 (C), 139.0 (C), 139.8 (C), 171.2 (C); MS (EI): m/z 91, 100% [C_6_H_5_CH_2_^+^], 279, 21% [M^+^]; Calcd for C_19_H_21_NO: C, 81.68; H, 7.58; N, 5.01. Found: C, 81.90; H, 7.61; N, 5.10. 

*3-Allyl-1,4,4-trimethyl-3,4-dihydroquinolin-2(1H)-one* (**6**): Yield 93%; yellowish solid; Mp 55–58 °C; ^1^H-NMR (CDCl_3_) δ: 1.24 (3H, s, CH_3_), 1.35 (3H, s, CH_3_), 1.96–2.01 (1H, m, CH), 2.27–2.32 (1H, m, CH), 2.44 (1H, dd, *J* = 10.2, 4.8 Hz, CH), 3.39 (3H, s, NCH_3_), 4.90–4.96 (2H, m, CH=C**H_2_**), 5.76–5.84 (1H, m, C**H**=CH_2_), 7.01 (1H, d, *J* = 7.2 Hz, H_ar_), 7.08 (1H, t, *J* = 7.5 Hz, H_ar_), 7.26–7.30 (2H, m, 2 × H_ar_); ^13^C-NMR (CDCl_3_) δ: 23.4 (CH_3_), 28.6 (CH_3_), 29.4 (CH_2_), 31.4 (CH_2_), 36.1 (C), 53.7 (CH), 114.8 (CH), 116.1 (CH_2_), 123.3 (CH), 124.8 (CH), 127.3 (CH), 133.6 (C), 136.2 (CH), 138.8 (C), 171.4 (C); MS (EI): m/z 214, 100% [M-CH_3_^+^], 229, 73% [M^+^]; Calcd for C_15_H_19_NO: C, 78.56; H, 8.35; N, 6.11. Found: C, 78.66; H, 8.41; N, 6.02.

*3-(5-Bromopentyl)-1,4,4-trimethyl-3,4-dihydroquinolin-2(1H)-one* (**7**): Yield 60%; yellowish oil; ^1^H- NMR (CDCl_3_) δ: 1.21 (3H, s, CH_3_), 1.34 (3H, s, CH_3_), 1.30-1.50 (6H, m, 3 × CH_2_), 1.80 (2H, m, CH_2_), 2.30 (1H, dd, *J* = 7.8, 3.9 Hz, CH), 3.37 (2H, t, *J* = 6.8 Hz, CH_2_), 3.40 (3H, s, NCH_3_), 7.00 (1H, d, *J* = 8.1 Hz, H_ar_), 7.08 (1H, td, *J* = 7.5, 1.2 Hz, H_ar_), 7.24-7.30 (2H, m, 2 × H_ar_); ^13^C-NMR (CDCl_3_) δ: 23.4 (CH_3_), 26.4 (CH_2_), 27.2 (CH_2_), 28.3 (CH_2_), 28.7 (CH_3_), 29.5 (CH_3_), 32.6 (CH_2_), 33.9 (CH_2_), 36.1 (C), 53.5 (CH), 114.8 (CH), 123.3 (CH), 124.8 (CH), 127.2 (CH), 133.8 (C), 138.9 (C), 172.1 (C); MS (EI): m/z 174, 100% [M-(CH_2_)_5_Br-CH_3_^+^], 337, 4% [M(Br^79^) ^+^], 339, 4% [M(Br^81^) ^+^]; Calcd for C_17_H_25_BrNO: C, 60.36; H, 7.15; N, 4.14. Found: C, 59.98; H, 7.21; N, 4.32.

*3-(Hydroxy(phenyl)methyl)-1,4,4-trimethyl-3,4-dihydroquinolin2(1H)-one* (**8**): Yield 99%; white solid; Mp 143–146 °C; ^1^H-NMR (CDCl_3_) δ: 1.11 (3H, s, CH_3_), 1.54 (3H, s, CH_3_), 2.80 (1H, d, *J* = 4.5 Hz, CH), 2.90 (3H, s, NCH_3_), 3.26 (1H, brs, O**H**), 4.75 (1H, d, *J* = 4.5 Hz, CH), 6.42 (1H, dd, *J* = 8.1, 1.5 Hz, H_ar_), 6.77–6.80 (2H, m, 2 × H_ar_), 6.87–6.98 (5H, m, 5 × H_ar_), 7.09 (1H, dd, *J* = 7.2, 1.8 Hz, H_ar_); ^13^C-NMR (CDCl_3_) δ: 24.1 (CH_3_), 29.1 (CH_3_), 30.5 (CH_3_), 35.5 (C), 61.2 (CH), 72.3 (CH), 114.4 (CH), 123.4 (CH), 124.3 (CH), 126.0 (2xCH), 127.1 (CH), 127.2 (2 × CH), 127.6 (CH), 132.9 (C), 138.2 (C), 140.9 (C), 171.1 (C); MS (EI, DI): m/z = 174, 100% [M-CH_3_-C_6_H_5_CHOH^+^], 295, 13% [M^+^] ; Calcd for C_19_H_21_NO_2_: C, 77.26; H, 7.17; N, 4.74. Found: C, 78.02; H, 7.23; N, 4.75.

*3-Iodo-1,4,4-trimethyl-3,4-dihydroquinolin-2(1H)-one* (**9**): Yield 88%; beige solid; Mp 128–136 °C; ^1^H-NMR (CDCl_3_) δ: 1.27 (3H, s, CH_3_), 1.47 (3H, s, CH_3_), 3.30 (3H, s, NCH_3_), 4.50 (1H, s, CH), 7.00 (1H, dd, *J* = 8.1, 0.9 Hz, H_ar_), 7.07 (1H, td, *J* = 7.2, 0.9 Hz, H_ar_), 7.18 (1H, dd, *J* = 7.8, 1.5 Hz, H_ar_), 7.25 (1H, td, *J* = 7.5, 1.8 Hz, H_ar_); ^13^C-NMR (CDCl_3_) δ: 23.2 (CH_3_), 28.3 (CH_3_), 30.1 (CH_3_), 37.4 (CH), 37.8 (C), 115.1 (CH), 123.9 (CH), 124.6 (CH), 127.9 (CH), 132.1 (C), 138.7 (C), 167.3 (C); MS (EI): m/z 144, 100% [M-I-CH_3_-NCH_3_^+^], 315, 74% [M^+^]; Calcd for C_12_H_14_INO: C, 45.73; H, 4.48; N, 4.44. Found: C, 46.03; H, 4.51; N, 4.47.

*3-Acetyl-1,4,4-trimethyl-3,4-dihydroquinolin-2(1H)-one* (**10**): Yield 68%; white solid; Mp 97–99 °C; ^1^H-NMR (CDCl_3_) δ: 1.29 (3H, s, CH_3_), 1.43 (3H, s, CH_3_), 2.17 (3H, s, COCH_3_), 3.43 (3H, s, NCH_3_), 3.59 (1H, s, CH), 7.03 (1H, dd, *J* = 8.4, 1.2 Hz, H_ar_), 7.11 (1H, td, *J* = 7.8, 1.2 Hz, H_ar_), 7.29 (2H, m, 2 × H_ar_). ^13^C-NMR (CDCl_3_) δ: 23.6 (CH_3_), 28.6 (CH_3_), 29.9 (CH_3_), 31.9 (CH_3_), 36.1 (C), 66.2 (CH), 115.0 (CH), 123.9 (CH), 124.0 (CH), 127.5 (CH), 133.4 (C), 138.5 (C), 166.2 (C), 203.0 (C); MS (EI): m/z 174, 100% [M-COCH_3_-CH_3_^+^], 231, 32% [M^+^]; Calcd for C_14_H_17_NO_2_: C, 72.70; H, 7.41; N, 6.06. Found: C, 72.43; H, 7.39; N, 6.11.

*3-Chloro-1,4,4-trimethyl-3,4-dihydroquinolin-2(1H)-one* (**11**): Yield 90%; yellowish solid; Mp 90–93 °C; ^1^H-NMR (CDCl_3_) δ: 1.20 (3H, s, CH_3_), 1.39 (3H, s, CH_3_), 3.35 (3H, s, NCH_3_), 4.17 (1H, s, CH), 6.98 (1H, dd, *J* = 8.1, 1.5 Hz, H_ar_), 7.06 (1H, td, *J* = 7.8, 1.2 Hz, H_ar_), 7.21–7.27 (2H, m, 2 × H_ar_); ^13^C-NMR (CDCl_3_) δ: 23.1 (CH_3_), 26.2 (CH_3_), 30.1 (CH_3_), 38.5 (C), 64.7 (CH), 115.2 (CH), 124.0 (CH), 125.3 (CH), 127.9 (CH), 131.2 (CH), 138.0 (C), 165.3 (C); MS (EI): m/z 208, 100% [M-CH_3_^+^], 223, 92% [M(Cl^35^) ^+^], 225, 31% [M(Cl^37^) ^+^]; Calcd for C_12_H_14_ClNO: C, 64.43; H, 6.31; N, 6.26. Found: C, 64.72; H, 6.49; N, 6.30.

*1,4,4-Trimethyl-3-(phenylsulfonyl)-3,4-dihydroquinolin-2(1H)-one* (**12**): Yield 46%; yellowish solid; Mp 110–114 °C; ^1^H-NMR (CDCl_3_) δ: 1.26 (3H, s, CH_3_), 1.90 (3H, s, CH_3_), 3.22 (3H, s, NCH_3_), 4.04 (1H, s, CH), 6.80 (1H, dd, *J* = 7.8, 0.9 Hz, H_ar_), 7.12 (1H, td, *J* = 7.5, 1.2 Hz, H_ar_), 7.21 (1H, td, *J* = 7.8, 1.5 Hz, H_ar_), 7.32 (1H, dd, *J* = 7.5, 1.5 Hz, H_ar_), 7.40 (2H, t, *J* = 7.8 Hz, 2 × H_ar_), 7.55 (1H, t, *J* = 7.5 Hz, H_ar_), 7.60–7.63 (2H, m, 2 × H_ar_); ^13^C-NMR (CDCl_3_) δ: 23.9 (CH_3_), 30.1 (CH_3_), 31.1 (CH_3_), 37.0 (C), 77.6 (CH), 115.1 (CH), 124.3 (CH), 124.4 (CH), 127.9 (CH), 128.6 (2 × CH), 128.7 (2 × CH), 131.3 (C), 133.9 (CH), 138.1 (C), 138.6 (C), 161.8 (C); MS (EI): m/z 250, 100%, 329, 57% [M^+^]; Calcd for C_18_H_19_NO_3_S: C, 65.63; H, 5.81; N, 4.25. Found: C, 64.84; H, 5.89; N, 4.20.

*1,4,4-Trimethyl-2-oxo-1,2,3,4-tetrahydroquinoline-3-carboxylic acid* (**13**): Yield 75%; Brown solid; Mp 138–140 °C; ^1^H-NMR (CDCl_3_) δ: 1.31 (3H, s, CH_3_), 1.35 (3H, s, CH_3_), 3.35 (3H, s, NCH_3_), 3.36 (1H, s, CH), 6.95 (1H, d, *J* = 8.1 Hz, H_ar_), 7.03 (1H, t, *J* = 7.2 Hz, H_ar_), 7.19–7.27 (2H, m, 2 × H_ar_), 10.25 (1H, brs, COOH); ^13^C-NMR (CDCl_3_) δ: 23.7 (CH_3_), 27.5 (CH_3_), 30.1 (CH_3_), 36.3 (C), 58.8 (CH), 115.3 (CH), 124.0 (CH), 124.1 (CH), 127.7 (CH), 133.3 (C), 138.4 (C), 166.2 (C), 172.7 (C); MS (EI, DI): m/z 174, 100% [M-CH_3_-COOH^+^], 233, 19% [M^+^]; Calcd for C_13_H_15_NO_3_: C, 66.94; H, 6.48; N, 6.00. Found: C, 66.87; H, 6.41; N, 6.13.

## 4. Conclusions

In summary, we have described in this paper the lithiation and electrophilic substitution of 1*,*4,4-trimethyl-3,4-dihydroquinolin-2(1*H*)-one in position 3 and the unexpected reaction with different sulfonyl derivatives under the same conditions, providing either a chlorinated or sulfonyl compound. The enantioselective substitution of this position is currently under investigation in our laboratory. The biological evaluation of these new compounds is also now under investigation.
